# The effects of long-term stress on neural dynamics of working memory processing: An investigation using ERP

**DOI:** 10.1038/srep23217

**Published:** 2016-03-22

**Authors:** Yiran Yuan, Ada W. S. Leung, Hongxia Duan, Liang Zhang, Kan Zhang, Jianhui Wu, Shaozheng Qin

**Affiliations:** 1 Key Laboratory of Behavioral Science, Institute of Psychology, Chinese Academy of Sciences, Beijing, 100101, China; 2 Department of Occupational Therapy, Faculty of Rehabilitation Medicine, University, Alberta, T6G 2G4, Canada; 3 Rotman Research Institute, Baycrest Centre for Geriatric Care, Ontario, Toronto, M6A 2E1, Canada; 4 University of Chinese Academy of Sciences, Beijing, 100049, China; 5 State Key Laboratory of Cognitive Neuroscience and Learning & IDG/McGovern Institute for Brain Research, Beijing Normal University, China; 6 Department of Psychiatry and Behavioral Sciences, Stanford University School of Medicine, Stanford, California, U.S.A

## Abstract

This study examined the neural dynamics of working memory (WM) processing under long-term stress. Forty participants who had been exposed to a long period of major exam preparation (six months) and twenty-one control participants performed a numerical n-back task (n = 1, 2) while electroencephalograms were recorded. Psychological and endocrinal measurements confirmed significantly higher levels of long-term stress for participants in the exam group. The exam group showed significantly increased P2 amplitude in the frontal-central sites in the 1-back and 2-back conditions, whereas other ERP components, including the P1, N1 and P3 and behavioral performance, were unchanged. Notably, the P2 effect was most pronounced in participants in the exam group who reported perceiving high levels of stress. The perceived stress scores positively correlated with the P2 amplitude in the 1-back and 2-back conditions. These results suggest that long-term stress has an impact on attention and the initiation of the updating process in WM.

Exposure to high levels of long-term and sustained stress has a variety of consequences on brain and cognition^1^. Among these consequences, long-term stress has been linked with impoverished higher-order cognitive prefrontal functions, such as attentional control and working memory (WM)^2^,^3^. Such an effect is thought to result from a complex interplay among perceived stress and prolonged activation of stress-sensitive neuromodulatory systems^4^,^5^.

A wealth of behavioral research has demonstrated significant WM deficits in humans under a variety of long-term stressors, such as early life stress^6^, caregiver stress^7^ and chronic stress in outpatients^8^. Some studies, however, reported that the long-term stress had a null effect on WM performance^9^,^10^. These different results on the effect of long-term stress on WM performance may due to variability in stressors, behavioral tasks and participants’ characteristics.

WM refers to the ability to maintain and manipulate information over a short period of time for goal-directed actions^11^. Neuroimaging studies on WM have reported activation in regions comprising the widespread attention network^12^,^13^. Within this attention network, both the prefrontal regions and posterior sensory cortices are thought to be responsible for maintaining image representation in WM^14^,^15^. The prefrontal region maintains representations of multiple goal-related information that serve to influence stimulus-specific activity in sensory regions, and the posterior sensory cortices maintain high-fidelity representations of WM content^15^. The parietal cortex plays a central role in monitoring updating processes and is activated differentially for workload and stimulus characteristics in WM^16^,^17^. Smith and Jonides (1998) have outlined a model describing various cognitive processes, such as attention and executive processes, in WM. According to the model, stimuli are first encoded and then translated into phonological representations that are rehearsed sub-vocally before appropriate actions are carried out^18^. Previous studies using functional magnetic resonance imaging (fMRI) have suggested that long-lasting psychosocial stress disrupts the attention and executive processes in the frontal-parietal attention network^10^,^19^. Some studies reported the effect of stress on the neural activities in the frontal-parietal network, though no noted behavioral decrement was found^9^,^10^. However, fMRI has a limited temporal resolution and may not elucidate how long-term stress affects different cognitive stages of WM processing.

The event-related potential (ERP) technique, which can provide high temporal resolutions in milliseconds, is an ideal method to examine alterations in the dynamic time course of neural activity during WM processing under long-term stress exposure. The P1 and N1 components are related to the early processing of stimuli and are sensitive to the physical properties of the stimuli^20^,^21^. These two components are modulated by attention and are generated in the extrastriate cortex for visual tasks^22^. Specifically, the P1 is thought to reflect sensory selection^23^, whereas the N1 indexes the orienting of attention^24^. The P2 component has been related to working memory processes^25^,^26^. Prior studies using a modified continuous performance task suggested that the P2 component reflects the onset of context updating in WM^27^. Other studies using n-back tasks to study WM found individual differences in the P2 but not earlier components such as the P1 and N1. For example, they found larger P2 amplitude in patients with liver cirrhosis than in the control group^28^ or participants in low arousal conditions than in high arousal conditions^29^. The P3 component is indicative of response selection and maintenance of the updating process in WM^27^. Some studies have shown that P3 amplitudes are sensitive to memory loads and decrease with increasing *n* in n-back tasks^30^.

In the past, some ERP studies have addressed the effect of stress on WM processing. Most of these studies have focused on patients with post-traumatic stress disorder (PTSD). For example, they found that PTSD patients showed smaller P3 amplitudes than healthy controls in n-back tasks^31^,^32^. To our knowledge, however, little is known about how long-term stress affects the neural dynamics associated with WM processing in otherwise healthy people.

The aim of the present study was to investigate the neural dynamics of WM processing under long-term stress by using n-back paradigms and ERP techniques. Forty healthy young adults who were exposed to a competitive Chinese National Postgraduate Entrance Exam (CNPEE) and twenty-one controls who were not exposed to the exam underwent ERP recordings while performing a numerical n-back WM task. The Perceived Stress Scale (PSS) and the cortisol awakening response (CAR) were obtained to assess the stress response. The CAR is an endocrine marker of the hypothalamic-pituitary-adrenocortical (HPA) axis in response to the demands of the upcoming day, and long-term major stress has been linked with altered CAR in humans^33^,^34^. We hypothesized that long-term stress would have an impact on the cognitive processes in WM. We also expected that stress would have a more severe effect on the P2 and/or P3 components than the P1 and N1 components. Specifically, if long-term stress impacted early processes such as the onset of updating, then we would expect an effect on the P2 component. Conversely, if the effect of stress was related more to judgment and memory load demands, then we would expect an effect on the P3 component.

## Results

### Psychological and endocrinal measurements of long-term stress

**Figure 1:**
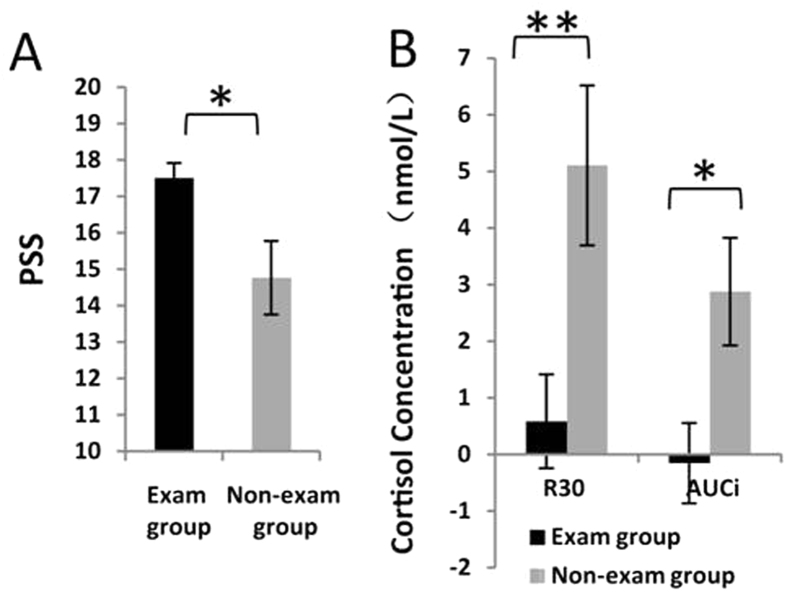
Average psychological and endocrinal measurements in the exam and non-exam groups. (**A**) PSS; (**B**) CAR, measured by R_30_ and AUC_i_, (nmol/L). Error bars represent the standard error of the mean. Note: ^∗^*p* < 0.05; ^∗∗^*p* < 0.01.

Descriptive statistics for the PSS and CAR parameters between the two groups are shown in Fig. 1.

The PSS in the exam group was significantly higher than the non-exam group (t = 2.506, df = 27, *p* = 0.019). The median score of PSS in the exam group was 17. Considering that the PSS is not a specific-population-dependent instrument^35^ and a score of 17 is higher than that in the community residents of the original norms^36^, we classified the eight participants who had a score of 17 into the high-stress exam group. Hence, the entire exam group was divided by a median split into a high-stress exam group (N = 24; M ± SD: 19.08 ± 2.06) and a low-stress exam group (N = 16; M ± SD: 15.13 ± 1.09).

The CAR parameters (R_30_ and AUC_i_) were significantly lower in the exam group than in the non-exam group (R_30_: t = −2.957, df = 59, *p* = 0.004; AUC_i_: t = −2.536, df = 59, *p* = 0.014).

No significant differences between the two groups on the Big Five factors of the Mini-IPIP were found (all |t|_s_ < 1.391, *p*_*s*_ > 0.1).

### Effects of long-term stress on WM performance

First, we examined the differences of WM performance between the exam and non-exam groups. The analysis of correct rate and reaction time (RT) showed a significant main effect for workload (1-back vs. 2-back). In comparison with the 2-back condition, participants had significantly higher correct rates and faster RTs in the 1-back condition (correct rate: F_(1,59)_ = 25.701, *p* < 0.001, partial η^2^ = 0.303; RT: F_(1,59)_ = 71.083, *p* < 0.001, partial η^2^ = 0.546). There were no significant differences in the main effect for group or the interaction between the two factors (*p*_*s*_ > 0.1).

**Figure 2:**
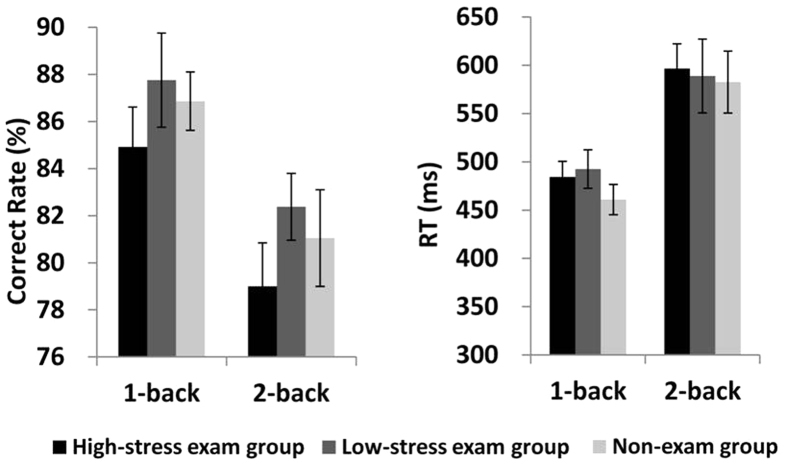
Correct rate and RT for the 1-back and 2-back tasks in the high-stress exam group, low-stress exam group and non-exam group. Error bars represent the standard error of the mean.

We then examined the difference of WM performance between three subgroups (high-stress exam group vs. low-stress exam group vs. non-exam group). The analysis of the correct rate and RT showed a significant main effect for workload (1-back vs. 2-back). In comparison with the 2-back condition, participants had significantly higher correct rates and faster RTs in the 1-back condition (correct rate: F_(1,58)_ = 26.704, *p* < 0.001, partial η^2^ = 0.315; RT: F_(1,58)_ = 70.714, *p* < 0.001, partial η^2^ = 0.549). There were no significant differences in the main effect for group or the interaction between the two factors (*p*_*s*_ > 0.1) (see Fig. 2).

### Effects of long-term stress on ERP data

#### P1 & N1

The results of the ANOVAs showed that there were neither significant main effects for workload and group (two groups: exam group vs. non-exam group or the three subgroups: high-stress exam group vs. low-stress exam group vs. non-exam group), nor interaction effects for workload × electrode, group × workload, and group × electrode on the P1 and N1 amplitudes respectively (*p*_*s*_ > 0.1).

#### P2

First, we examined the differences of P2 amplitudes between the two groups. The ANOVA showed a significant main effect for workload (F_(1,59)_ = 18.552, *p* < 0.001, partial η^2^ = 0.239), with a larger P2 amplitude in the 2-back condition. Most importantly, we found a significant main effect for group on the P2 amplitude. The exam group had a significantly larger P2 amplitude than the non-exam group (F_(1,59)_ = 4.606, *p* = 0.036, partial η^2^ = 0.072). The interaction effects for workload × sagittal row, workload × laterality, group × workload, group × sagittal row, and group × laterality did not reach significance (*p*_*s*_ > 0.1).

**Figure 3:**
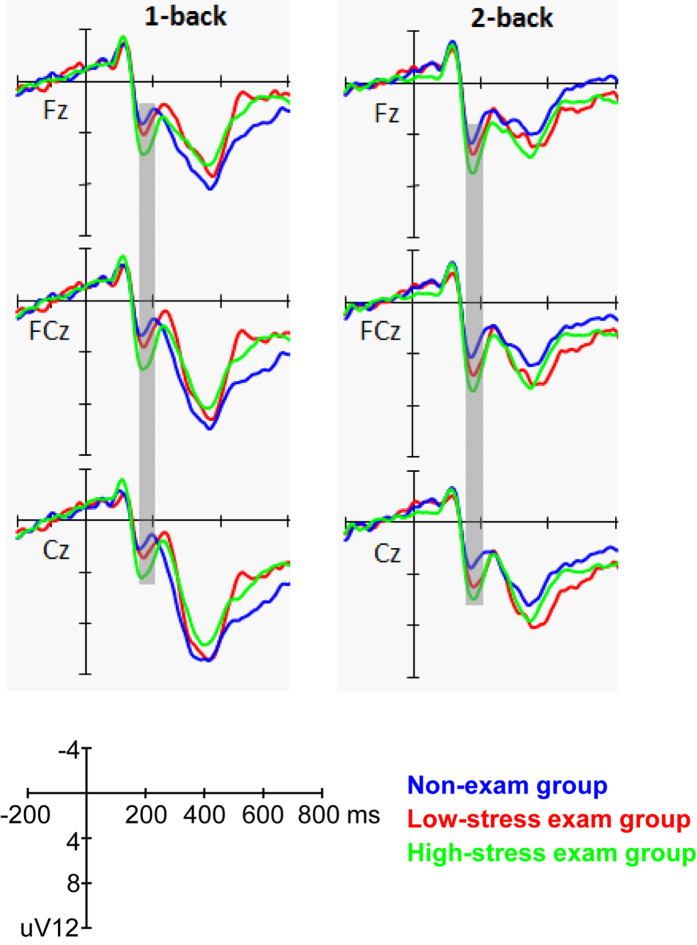
Grand average ERPs elicited by performing the 1-back and 2-back tasks in the high-stress exam group, low-stress exam group and non-exam group at the midline electrodes. The gray areas highlight the time window for P2 (160–210 ms) that was used for the statistical analysis.

**Figure 4:**
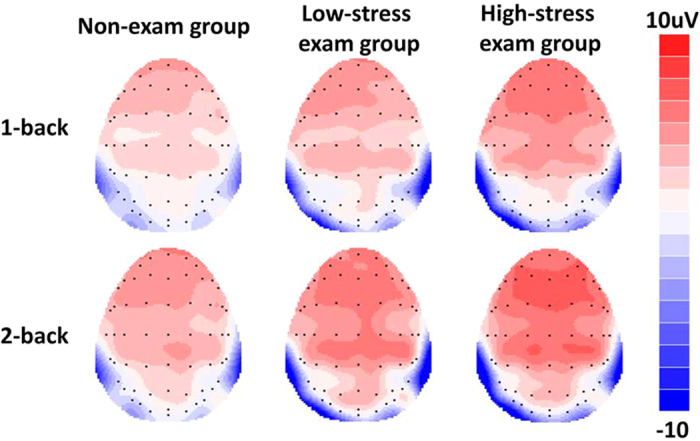
Grand average ERP topographies of the P2 component (160–210 ms) across different conditions.

We then examined the differences of P2 amplitudes between three subgroups (high-stress exam group vs. low-stress exam group vs. non-exam group). The ANOVA showed a significant main effect for workload (F_(1,58)_ = 20.165, *p* < 0.001, partial η^2^ = 0.258), with a larger P2 amplitude in the 2-back condition. The interaction effects for workload × sagittal row and workload × laterality reached significance (F_(2,116)_ = 3.932, *p* = 0.044, partial η^2^ = 0.063 and F_(2,116)_ = 3.293, *p* = 0.049, partial η^2^ = 0.054, respectively). Post-hoc comparisons revealed that the effect of workload on P2 amplitudes was maximal at the frontal (F) row and midline electrodes. The main effect for group was marginally significant (F_(2,58)_ = 3.046, *p* = 0.055, partial η^2^ = 0.095). A post-hoc LSD test showed that only the high-stress exam group had a significantly larger P2 amplitude than the non-exam group (*p* = 0.017), while no other differences reached significance (*p*_*s*_ > 0.1). The interaction effects for group × workload, group × sagittal row, and group × laterality did not reach significance (*p*_*s*_ > 0.1) (see Figs 3 and 4).

#### P3

We examined the differences in P3 amplitudes in terms of workload, sagittal row and laterality, as well as group factors. The ANOVA showed a significant main effect for workload (F_(1,59)_ = 26.959, *p* < 0.001, partial η^2^ = 0.314), with a larger P3 amplitude in the 1-back condition. The interaction effects for workload × sagittal row and workload × laterality reached significance (F_(2,118)_ = 6.865, *p* = 0.005, partial η^2^ = 0.104 and F_(2,118)_ = 6.445, *p* = 0.003, partial η^2^ = 0.098, respectively). Post-hoc comparisons revealed that the effect of workload on P3 amplitudes was maximal at the centro-parietal (CP) row and midline electrodes. The main effect for group was not significant (F_(1,59)_ = 0.353, *p* = 0.555, partial η^2^ = 0.006). In addition, the interaction effects for group × workload, group × sagittal row, and group × laterality did not reach significance (*p*_*s*_ > 0.1).

**Figure 5:**
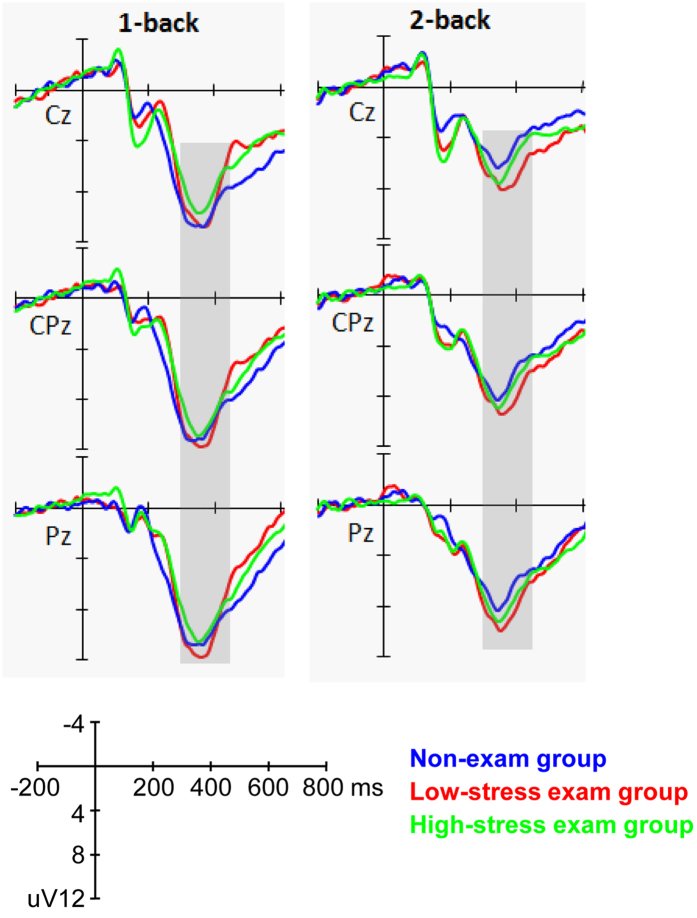
Grand average ERPs elicited by performing the 1-back and 2-back tasks in the high-stress exam group, low-stress exam group and non-exam group at the midline electrodes. The gray areas highlight the time window for P3 (300–450 ms) that was used for the statistical analysis.

When subdividing the exam group into high-stress and low-stress sub-groups, the ANOVA results were similar for the two groups. The main effect for workload was significant (F_(1,58)_ = 22.893, *p* < 0.001, partial η^2^ = 0.283), with a larger P3 amplitude in the 1-back condition. The interaction effects for workload × sagittal row and workload × laterality reached significance (F_(2,116)_ = 5.486, *p* = 0.013, partial η^2^ = 0.086 and F_(2,116)_ = 5.033, *p* = 0.009, partial η^2^ = 0.080, respectively). Post-hoc comparisons revealed that the effect of workload on P3 amplitudes was maximal at the centro-parietal (CP) row and midline electrodes. The main effect for group (high-stress exam group vs. low-stress exam group vs. non-exam group) was not significant (F_(2,58)_ = 0.448, *p* = 0.641, partial η^2^ = 0.015). In addition, the interaction effects for group × workload, group × sagittal row, and group × laterality did not reach significance (*p*_*s*_ > 0.1) (see Fig. 5).

### Correlation analyses

For the entire participant sample, the relationship between the PSS and the P2 amplitude at the Fz electrode was significant in both the 1-back and 2-back conditions (1-back: *r* = 0.328, *p* = 0.010; 2-back: *r* = 0.301, *p* = 0.018). No significant relationships between the PSS and behavioral performance (correct rate and RT) or other ERP components were found (*p*_*s*_ > 0.1).

For the entire participant sample, the relationship between the P1 and N1 amplitudes and the behavioral performance (correct rate and RT) was not significant (*p*_*s*_ > 0.1). The correlation between the P2 amplitude and RT was significant in the 2-back condition (*r* = −0.253, *p* = 0.049), but not significant in the 1-back condition (*r* = −0.145, *p* = 0.264). The P3 amplitude was also negatively correlated with RT in both the 1-back and 2-back conditions (1-back: *r* = −0.344, *p* = 0.007; 2-back: *r* = −0.390, *p* = 0.002).

## Discussion

The present study examined the effects of long-term stress on the neural dynamics of WM processing with n-back tasks in healthy adults. The PSS confirmed significantly higher levels of long-term stress for participants in the exam group. Endocrinal results revealed a significantly decreased CAR in the exam group compared with the non-exam group^37^, which provides endocrinal index for long-term stress.

As expected, our results showed no group differences in the P1 and N1 components. The P1 component is thought to reflect sensitivity to the physical characteristics of exogenous stimuli^38^, while the N1 component is associated with perceptual processes at the early stage of visual information processing^23^,^39^. Hence, our results suggested that the neural activity was comparable during the stimulus encoding stage of WM processing between the groups. Our results showed that there was a smaller P2 and a larger P3 component in the 1-back than in 2-back condition in both groups; these were consistent with previous studies which found that an increase in the demands of working memory load results in an increase in P2 and a reduction of P3 amplitude^30^,^40^. Additionally, our results from the correlation analysis revealed that the amplitudes of P2 and P3 components, but not the P1 and N1 components, was significantly correlated with the behavioral performance of the n-back task, further suggesting that neural activity of the onset and maintenance of context updating in WM is more related with the behavior performance of WM. Therefore, we focused our discussion on the P2 and P3 components.

Our results revealed that long-term stress led to an increase in P2 amplitudes at the frontal-central sites when participants performed the 1-back and 2-back tasks. Previous studies have found that the P2 component reflects an early stage of information processing, one of the core stages in many cognitive tasks, including the n-back task^28^,^41^. Recently, some studies have found that the P2 component is indicative of the initial stage of context updating, whereas subsequent components such as the P3 and late positive components are indicative of continuous monitoring of the updating process^27^. During WM, the onset of updating occurs after stimuli are encoded and translated to phonological representations. Therefore, this early stage of updating is one of the crucial steps for successful task performance. Our results are consistent with this viewpoint as we found significant correlations with behavioral responses on the P2 and P3 amplitudes. In addition, the literature has also suggested that the P2 component is associated with directing attentional resources to stimuli^42^,^43^. Some previous ERP studies have detected alterations in P2 components but not in P3 components in people with social anxiety disorders^44^,^45^. Based on the neurocognitive models of anxiety, attention bias is thought to be a key feature of cognitive dysfunction in a variety of anxiety-related disorders, most likely due to sustained anxiety and long-term or chronic stress exposure. Taken all together, our results on the increased P2 amplitude in high stress individuals probably suggest that long-term stress has an impact on the cognitive stages involving attention and the onset of updating in WM. Furthermore, group comparisons indicated that the P2 amplitude was most pronounced for participants in the exam group who reported high levels of perceived stress, and correlation analysis showed that the P2 amplitude was positively correlated with the PSS. This suggests that the self-perceived stress plays a critical role in the modulation of P2 components for individuals who are under long-term stress.

Although the stress group had a greater P2 amplitude than the non-stress group, the P3 amplitude and the final behavioral performance in the present study remain unchanged. One possible explanation for the lack of P3 and behavioral differences in our study may be that the type of task used, i.e., the 1-back and 2-back tasks, were too simple, causing a ceiling effect that obscures small differences in the P3 component and final behavioral performance. Nevertheless, the behavioral results, i.e., long-term stress has no significant effect on WM performance, were consistent with those of previous studies^9^,^10^. Future research using more difficult WM tasks, e.g., 3-back or 4-back, may be needed to verify this interpretation. Another explanation for our results is that the effects of long-term stress may have selectively impacted neural processes originating in the prefrontal regions. According to the guided activation theory proposed by Miller and Cohen (2001), the neural activity in the prefrontal cortex represents task context and guides posterior activities for task execution^46^. Since the P2 component is most prominent at the frontal-central sites covering part of the prefrontal cortex, as opposed to the P3 which dominates at the parietal sites, any changes in neural activities of the prefrontal regions would more readily be reflected on the P2 than the P3 component.

Recently, research has proposed that working memory might be better conceptualized as a limited memory resource that is distributed flexibly among all items to be maintained in memory, including the events and objects occurring in the n-back task^47^. This new conceptualization is based on the findings that decline in precision of task performance is associated with increasing working memory load and varied precision costs are observed for stimuli of different salience^48^,^49^. Our results appear in line with the limited memory resource account as participants’ memory resource might have been jeopardized due to prolonged exposure to stress, causing more effort in processing and updating the stimulus and resulting in greater P2 amplitudes. Although the difference in the P3 component between the two groups was not prominent from our results, there were significant relationships between the behavioral performance and the P3 amplitudes. This suggested that changes of load related brain activity (the P3 amplitude) are modulated by how well the participants perform the task. Future study would need to verify the role of P3 components in long-term stress exposure by utilizing different WM tasks and tasks of higher difficulty levels.

The ERP results obtained from the present study have important implications for the effects of long-term stress on the neural dynamics of n-back WM tasks. With the advantage of high temporal resolution in ERPs, we were able to examine different cognitive processes in WM. In addition, our results imply that when we evaluate the influence of stress on cognitive performance, we cannot depend solely on behavioral output. Notably, before stress explicitly affects behavior, change(s) at the processing stage(s) preceding the behavioral response may have already occurred. Our results also coincided with previous fMRI studies in that we found no significant effect on behavioral performance, but did find a significant effect of stress on the frontal-parietal network^9^,^10^.

Our study has some limitations that must be addressed. First, we included only male undergraduate students in the study, and the results might not be generalized to female students. Second, we did not conduct a longitudinal study to investigate how psychological and electrophysiological responses change over time. Although we controlled some factors that could influence the characteristics of the two groups, such as personality traits, the homogeneity of the participants and other major life stressors (i.e., economic problem), there may still be other variables that differentiate the two groups. Future studies can consider adding a test during the non-exam period to observe a baseline for studying the effects of stress imposed by the exam.

In conclusion, the results of our study suggest that the effects of long-term stress on WM processing occur at the level of cognitive processes involving attention and the onset of updating, as indicated by the P2 component. In addition, the effects of long-term stress do not seem to manipulate the later and maintenance stage of the updating process in WM (as indicated by P3 component) and the final behavioral performance. Future study would need to employ more demanding tasks to verify the role of P3 components in long-term stress exposure.

## Method

### Participants

We chose the CNPEE as the long-term stressor in this study. The CNPEE is an important and competitive exam in the Chinese educational system; more than two-thirds of examinees have failed the exam each year over the last ten years^50^. Generally, examinees require approximately six months to prepare for the exam. Previous studies have also established examination preparation as a long-term stressor^3^,^51^. Therefore, the CNPEE is a perfect candidate for studying the effect of long-term stress on WM.

Sixty-three young, healthy undergraduate students were recruited through advertisement. Because of gender differences on stress response^52^, only male participants were included in the present study. The participants had no history of self-reported serious diseases (i.e., no psychiatric, neurological or endocrine disorders, no chronic physiological disorders and no serious trauma), no current diseases (i.e., acute inflammation, allergy, acute episodes of chronic disease or periodontitis), no current medication use within two days of participation in the study, no excessive alcohol use (more than two alcoholic drinks daily) or nicotine consumption (more than five cigarettes a day), and a regular living style (i.e., no irregular circadian rhythms).

To exclude the possible differences between the two groups, we assessed their personality trait scores as mentioned below (see *Psychological measurements* section). Moreover, all participants were chosen from the same medicine major within the same university, which suggested that there was no difference between the two groups in the area of study. In addition, the participants in the non-exam group had not taken part in any major exam/interview within one month before and after participation in the study and had not experienced any major stressors during the past month, such as economic problems and interpersonal conflicts, as assessed by the Chinese version of the Life Events Scale (LES)^53^,^54^. The participants in the exam group were also given the LES to ensure that they were not exosed to any other major stressors.

All participants provided written informed consent and were paid for their participation. The experiment was approved by the Ethics Committee of Human Experimentation at the Institute of Psychology, Chinese Academy of Sciences. The methods were carried out in accordance with relevant guidelines and regulations. Two participants in the exam group were excluded due to poor behavioral performance (the correct rates were below three standard deviations from the norm level). Finally, forty participants in the exam group and twenty-one participants in the non-exam group were included in the data analysis. The exam and non-exam groups were matched for age (M ± SD: exam group 22.5 ± 1.0 years vs. non-exam group 22.6 ± 1.1 years).

### General procedure

This study was part of a project addressing the relationship between long-term stress and CAR/cognition^37^,^55^. All participants came to the laboratory between 11 and 25 days before the CNPEE. First, they completed some questionnaires as described below. After that they did several psychological tests, including a WM task while EEG data were recorded. Next, each participant was given eight saliva collection devices with detailed instructions about the method of saliva collection. On the next two consecutive days, the participants collected saliva samples by themselves and returned them to the laboratory as soon as possible.

### Psychological measurements

We used the Perceived Stress Scale (PSS) (10-item version)^36^ to assess the chronic stress level of each participant. The PSS has been frequently used for perception of chronic stress^3^,^56^. In the Chinese population, the PSS has been demonstrated to be a valuable standardized measure of psychological stress with high reliability and validity^35^,^57^. The Chinese version of the Mini International Personality Item Pool (the Mini-IPIP), which measures the Big Five factors of personality, including 20-items with the domains of neuroticism, extraversion, conscientiousness, openness, and agreeableness^58^,^59^ was also administered.

### Salivary cortisol sampling and analyses

Procedures for the sampling of saliva and collection of CAR have been reported in Duan *et al.*, (2013). Participants were instructed to collect saliva samples using Salivette collection devices (Sarstedt, Germany) immediately upon awakening (i.e., 0 min), 15, 30 and 60 min thereafter on each of the two consecutive days just after the day of the experiment. Each participant collected four samples per day, for a total of eight samples. After participants had collected the saliva samples, they were required to return them to the laboratory. The saliva samples were kept frozen (−20 °C) in the refrigerator until they were assayed. After thawing and centrifuging the saliva samples at 3200 rpm for 10 min, they were analyzed by electrochemiluminescence immunoassay (ECLIA, Cobas e 601, Roche Diagnostics).

### WM task

A numerical n-back task was used in the present study. Task difficulty was varied using two workloads (1-back vs. 2-back). Before the experiment, the participants took part in a practice session and received feedback. The experiment did not begin until the participants reached an accuracy rate above 80% in the practice session^29^. During the experiment, all of the participants took part in the 1-back task followed by the 2-back task without feedback. A series of one-digit numbers (from 1 to 9) in a random sequence was shown to the participants at a visual angle of approximately 1° horizontally and 2° vertically in the center of the computer screen. The color of the stimuli was white, and the background was black. Each digit was displayed for 500 ms with an inter-stimulus interval of 1700 ms. Participants were asked to respond by pressing the “match” button with the index finger of one of their hands when the number appeared on the screen was the same as the one presented one trial back for 1-back tasks and two trials back for 2-back tasks. The participants were also asked to respond by pressing the “non-match” button with the index finger of another hand if the presented number did not meet the “match” criterion. The match/non-match buttons were counterbalanced for the left/right hand across the participants. The participants were instructed to press the buttons as quickly and accurately as possible. Each of the 1-back and 2-back tasks consisted of 100 stimulus trials, with 50 match trials and 50 non-match trials. The trials were organized into four categories according to participants’ performance: 1-back correct, 1-back incorrect, 2-back correct and 2-back incorrect. In order to ensure the ERPs were not contaminated by error-related negativity, only the correct conditions were analyzed in the present study^60^. E-Prime software (Version 2.0, Psychology Software Tools, Inc., Pittsburgh, PA) was used to present the stimuli and record behavioral data. The experiment lasted about ten minutes.

### ERP recordings

Participants were seated in a normally illuminated room. During the WM task, electroencephalograms (EEG) were recorded continuously from 64 scalp locations which were distributed according to the international 10–20 system using Ag/AgCl electrodes (Neuroscan Inc., USA), with an on-line reference to the left mastoid. Signals were re-reference to the average of the left and right mastoid through off-line algebraic computations. Vertical eye movements were monitored by placing electrodes at 1 cm from the outer canthi of each eye, while the horizontal eye movements were monitored by placing electrodes above and below the left eye. Impedance was kept below 5 kΩ. The EEG signals were amplified by a Neuroscan SynAmps^2^ amplifier (Neuroscan Inc., USA) with a bandpass filter of 0.05–100 Hz and digitized at 1000 Hz.

The EEG data were processed offline by Scan 4.3 software (Neuroscan, USA). Ocular artifacts were removed from the EEG signals using a regression procedure built into the Neuroscan software^61^. The data were digitally lowpass filtered with 30 Hz, and were epoched into periods of 1000 ms (including a 200 ms pre-stimulus baseline) time-locked to the onset of stimuli. Trials with artifacts were rejected using a criterion of ±100 μV.

### Data analyses

We used independent *t*-tests to compare the differences between the exam and the non-exam group on all psychological measurements and CAR parameters. The results of CAR had been reported in an earlier study about the relationship between long-term stress and CAR^37^. In this study, we reported the CAR results again as two participants were excluded due to poor behavioral performance in the n-back tasks. The CAR consists of R_30_ and AUC_i_, which reflect cortisol levels (R_30_ was the change in the cortisol level 30 min after awakening; AUC_i_ was the area under the curve with respect to the increase of cortisol level; for more details, see Duan *et al.*, 2013). To examine the effects of individual differences on the psychological response to exam stress, the exam group was further divided by a median split into high- or low-stress exam groups based on their PSS.

For n-back performance, the correct rate and RT were calculated separately for the 1-back and 2-back tasks. Trials with incorrect responses or responses faster than 100 ms or slower than 2000 ms were excluded from behavioral and ERP analyses. Repeated-measures ANOVAs with workload (1-back vs. 2-back) as the within-subjects factor and group as the between-subjects factor were calculated separately for the correct rate and RT.

For the ERP data, we analyzed four components which occurred before behavioral response, namely, the P1, N1, P2 and P3, across different conditions. Therefore, the mean amplitude of P1 component was measured at O1, Oz and O2 (time window: 100–130 ms). The mean amplitude of N1 component was measured at PO5, PO6, PO7 and PO8 (time window: 160–180 ms). The mean amplitude of P2 component was measured during the time interval from 160 to 210 ms at nine electrodes: F3, Fz, F4, FC3, FCz, FC4, C3, Cz and C4. The mean amplitude of P3 was measured during the time interval from 300 to 450 ms at another nine electrodes: C3, Cz, C4, CP3, CPz, CP4, P3, Pz and P4. These electrodes were chosen as the amplitudes were largest at those sites. The amplitudes of P1 and N1 components were analyzed, respectively, with a repeated-measure ANOVA with workload and electrode (O1, Oz and O2 for P1 and PO5, PO6, PO7 and PO8 for N1) as within-subjects factors and group as the between-subjects factor. The amplitudes of P2 and P3 components were analyzed, respectively, with a repeated-measure ANOVA with workload and sagittal row (F, FC and C for P2 and C, CP and P for P3) and laterality (left, midline and right electrodes) as within-subjects factors and group as the between-subjects factor.

Pearson’s correlation analyses were conducted between the PSS and different dependent variables related to WM processing (including behavioral performance and ERP data). To assess the relationship between ERP components and WM processes, we conducted correlation analyses between ERP amplitudes and behavioral performance (correct rate and RT). Amplitudes for the P1, N1, P2 and P3 components extracted at O1, PO7, Fz and CPz, respectively, were used in the correlation analysis, as the amplitudes were largest at those sites.

We used the Greenhouse-Geisser correction to compensate for sphericity violations. When the ANOVAs revealed a significant main effect, the post-hoc Least Squared Difference (LSD) test was used to examine the specific effects and significance levels. Effect sizes were showed using eta square (partial η^2^). All reported *p* values were two-tailed.

## Additional Information

**How to cite this article**: Yuan, Y. *et al.* The effects of long-term stress on neural dynamics of working memory processing: An investigation using ERP. *Sci. Rep.* **6**, 23217; doi: 10.1038/srep23217 (2016).
